# How Life Works—A Continuous Seebeck-Peltier Transition in Cell Membrane?

**DOI:** 10.3390/e22090960

**Published:** 2020-08-30

**Authors:** Umberto Lucia, Giulia Grisolia

**Affiliations:** Dipartimento Energia “Galileo Ferraris”, Politecnico di Torino, Corso Duca degli Abruzzi 24, 10129 Torino, Italy; giulia.grisolia@polito.it

**Keywords:** life, non-equilibrium thermodynamics, heat and ions fluxes, what is life

## Abstract

This paper develops a non-equilibrium thermodynamic approach to life, with particular regards to the membrane role. The Onsager phenomenological coefficients are introduced in order to point out the thermophysical properties of the cell systems. The fundamental role of the cell membrane electric potential is highlighted, in relation to ions and heat fluxes, pointing out the strictly relation between heat exchange and the membrane electric potential. A Seebeck-like and Peltier-like effects emerge in order to simplify the description of the heat and the ions fluxes. Life is described as a continuos transition between the Peltier-like effect to the Seebeck-like one, and *viceversa*.

## 1. Introduction

Biology is the science of life, but, up to today, biologists do not agree on what life is, even if a great number of ways has been introduced to define it.

It is usual to avoid a direct definition of life, but to introduce a list of features based on:

- What it has: a cell, the basic unit of life, able to generate biochemical processes;
- What it does: growth, reproduction, adaptation and metabolism.

In this Communication, we wish to introduce a non-equilibrium thermodynamic approach to describing how life works and to introduce a different viewpoint based on thermoelectric effects (Peltier and Seebeck). Our aim is to develop this approach in the near future in order to improve our previous results on cancer [1], to support physicians, biologists, scientists and engineering in their research for new effective anticancer therapies. Here, we introduce the Onsager thermodynamic approach to life, with the aim of pointing out the thermophysical basis of life.

## 2. Materials and Methods

The living cell membrane is characterized by different permeabilities in relation to the distinct ions (Na${}^{+}$, K${}^{+}$, Cl${}^{-}$, Ca${}^{2+}$, etc.), which cause an electric potential difference Δϕ between the cytoplasm and the extracellular environment, measured in reference to the environment [2]. The membrane electric potential can be theoretically described by the Goldman-Hodgkin-Katz equation, which relates this potential to the permeability *P* and the concentrations of major ions present on both sides of the membrane itself, but also to the temperature [3,4]:

(1) $$\Delta \phi =\frac{RT}{F}\;\ln\left(\frac{P_{{\mathrm{Na}}^{+}}{\left[{\mathrm{Na}}^{+}\right]}_{\mathrm{outside}}+P_{K^{+}}{\left[K^{+}\right]}_{\mathrm{outside}}+P_{{\mathrm{Cl}}^{-}}{\left[{\mathrm{Cl}}^{-}\right]}_{\mathrm{outside}}}{P_{{\mathrm{Na}}^{+}}{\left[{\mathrm{Na}}^{+}\right]}_{\mathrm{inside}}+P_{K^{+}}[K_{\mathrm{inside}}^{]}+P_{{\mathrm{Cl}}^{-}}{\left[{\mathrm{Cl}}^{-}\right]}_{\mathrm{inside}}}\right),$$

where [A] is the concentration of the ion A, R=8.314 J mol${}^{-1}$K${}^{-1}$ is the universal constant of ideal gasses, *T* is the absolute temperature, *F* is the Faraday constant, and *P* is the relative permeability, such that $P_{{\mathrm{Na}}^{+}}=0.04$, $P_{K^{+}}=1$ and $P_{{\mathrm{Cl}}^{-}}=0.45$. But, the membrane potential can also be related to thermophysical and chemical quantities, with particular regard to the Gibbs energy variation and the pH variation, as follows [5]:

(2) ΔG=FΔϕ−2.3RTΔ(pH),

where *G* is the Gibbs energy, *F* is the Faradays constant, and 2.3Δ(pH) is the physiological concentration gradient.

In order to develop a non-equilibrium thermodynamic analysis, the general phenomenological relations must be introduced [6,7]:

(3) $$\left\{\begin{array}{c} J_{e}=-L_{11}\;\frac{\nabla \phi }{T}-L_{12}\;\frac{\nabla T}{T^{2}}\\ J_{Q}=-L_{21}\;\frac{\nabla \phi }{T}-L_{22}\;\frac{\nabla T}{T^{2}}, \end{array}\right.$$

where $J_{e}$ is the current density [A m${}^{-2}$], $J_{Q}$ is the heat flux [W m${}^{-2}$], *T* is the living cell temperature and $L_{ij}$ are the phenomenological coefficients, such that [8] $L_{12}=L_{21}$ in the absence of magnetic fields, and $L_{11}\geq 0$ and $L_{22}\geq 0$, and [8] $L_{11}L_{22}-L_{12}^{2}>0$. Considering that, in the stationary state, the net ions fluxes are null, in order to maintain constant both the membrane potential and the pH, it follows that $J_{e}=0$. Consequently, the previous equations holds to:

(4) $$\left\{\begin{array}{c} \frac{\nabla T}{T}=-\frac{L_{11}}{L_{12}}\;\nabla \phi \\ J_{Q}=\left(L_{22}\frac{L_{11}}{L_{12}}-L_{12}\right)\;\frac{\nabla \phi }{T}. \end{array}\right.$$

So, this is just a Seebeck-like effect [6] in the cell membrane. The second equation suggests a biophysical relation between the heat flux, through the cell membrane, and the membrane electric potential gradient.

Now, we consider that:

(5) $$\dot{Q}=\int_{A}J_{Q}\cdot \hat{n}\;dA,$$

where *A* is the area of the membrane external surface. Consequently, it is possible to write:

(6) $$\delta \dot{Q}=\left(L_{22}\frac{L_{11}}{L_{12}}-L_{12}\right)\;\frac{\nabla \phi }{T}\cdot \hat{n}\;dA=\frac{k}{T}\;\nabla \phi \cdot \hat{n}\;dA,$$

where $k=\left(L_{22}L_{11}/L_{12}\right)-L_{12}$ is a thermoelectric property of the cell, which links the heat flux to the membrane electric gradient. The Equation (6) represents a link between the heat power, exchanged between the cell and the environment, and the membrane potential gradient. Now, we consider that living cells exchange heat power with their environment, by the motion of the fluids (blood, water, etc.) around the cells themselves. This mechanism of heat exchange with fluids is thermodynamically described by convection, by the Newton law:

(7) $$\delta \dot{Q}=-\alpha \;\left(T-T_{0}\right)\;dA,$$

where $\rho \approx 10^{3}$ kg m${}^{-3}$ is the cell density, c≈4186 J kg${}^{-1}$ K${}^{-1}$ is the specific heat of the cell, $\alpha \approx 0.023Re^{0.8}Pr^{0.35}\lambda /\langle R\rangle$ is the coefficient of convection, with λ≈0.6 W m${}^{-1}$K${}^{-1}$ conductivity, Re≈0.2 the Reynolds number and Pr≈0.7 the Prandtl number [9], *A* area of the cell membrane, *V* is the cell volume, and 〈R〉=dV/dA≈V/A is the mean radius of the cell [10,11]. So, we can obtain:

(8) $$\frac{\partial \phi }{\partial \ell }=-\frac{\alpha }{\left(L_{22}\frac{L_{11}}{L_{12}}-L_{12}\right)}\;T\;\left(T-T_{0}\right)=-\frac{\alpha }{k}\;T\;\left(T-T_{0}\right),$$

which highlights the relation between the membrane electric potential and the temperature of the cell, *ℓ* being the length of the membrane. Moreover, this relation also points out the link between the gradient of the membrane electric potential, and the difference between the cell temperature *T* and its environmental temperature $T_{0}$. For a cell in a tissue, it is probable that the temperature of the cell is equal to that of the tissue—in this case, *T* is the temperature of the tissue (and of any cell in the tissue), and $T_{0}$ is an environmental temperature [12], which can be considered the temperature of the flowing blood for the more internal tissues, or of the environment of the body for the border tissues or for the body itself. In this case, if the environmental temperature increases up to a value greater than the temperature of the tissue (or of the body), some other biophysical mechanisms occur, in order to support the tissue to cool down.

In this way, following the considerations on life developed by Schödinger [12], we can state that life requires that always $T-T_{0}>0$, and, consequently, dϕ/dr<0. This last inequality highlights that the voltage difference between the cytoplasm and the extracellular environment is more negative in relation to the environment—the normal condition for life is that the cell membrane is hyperpolarized, which proves theoretically the experimental results [13,14,15,16].

If the current density persists to be null in time, the cell is not able to generate energy by its metabolism, and its temperature will decrease towards the temperature of its environment; consequently, the temperature difference results in ΔT≈0 °C. But, in this case, the cell will die [12]. So, the cell needs to exchange metabolites and ions, which analytically means that $J_{e}\neq 0$, such that [6,7]:

(9) $$\frac{dc_{i}}{dt}=-\nabla \cdot J_{i},$$

where $c_{i}$ is the concentration of the *i*-th ion (Na${}^{+}$, K${}^{+}$, Ca${}^{2+}$, Cl${}^{-}$, etc.), *t* is the time and $J_{i}$ is the current density of the *i*-th ion. In this condition, considering Equation (3), it follows the Thomson’s Second Relation [6,7]:

(10) $$\frac{d\phi }{dT}=-\frac{L_{21}}{L_{11}}\;\frac{1}{T}.$$

But, in this way, a Peltier-like effect occurs [6], and a temperature variation is generated by the variation of the membrane electric potential, due to the ions fluxes. But, a heat flux is also generated [6,7]:

(11) $$\frac{du}{dt}=-\nabla \cdot J_{u},$$

where *u* is the specific internal energy.

The results obtained can be summarised by stating that cell life cycle consists of [12]:

- A continue metabolic generation, characterised by ions and metabolites fluxes, for which a Peltier-like effect occurs and $d\phi /dT=-L_{21}/L_{11}T$
- A continued heat exchange, towards the environment, for which a Seebeck-like effect occurs and $\partial \phi /\partial \ell =-\alpha \;T\;(T-T_{0})/k$.

## 3. Results

The Seebeck-like and Peltier-like effects are two phenomena that occur in the cell membrane related to the ions and heat fluxes, fundamental for cell life. Indeed, ions and metabolites fluxes are related to the biochemical reactions, while heat fluxes to the thermodynamic condition for life to occur. So, during its life cycle, the cell membrane continues transitions between Seebeck-like and Peltier-like effects, in order to allow fluxes to occur in relation to heat exchange (Seebeck-like effect) and ions exchange (Peltier-like effect).

This result highlights that life consists of a continued membrane transition from Seebeck-like to Peltier-like effects and from Peltier-like to Seebeck-like effects. During the Seebeck-like effect, the membrane exchanges heat towards the environment. During the Peltier-like effect, the membrane exchanges ions, metabolites, and waste molecules with the environment.

## 4. Discussion and Conclusions

In 1969, Cone Jr. found that the start of the M phase of the cell cycle is characterised by a hyperpolarisation state, introducing the hypothesis that there exists a relation between the cell cycle progression and the membrane electric potential changes [17]. In 1970, Cone Jr. showed that membrane hyperpolarization is able to reversibly block the synthesis of DNA and the mitosis [18]. Last, in 1971, starting from experimental results [13,14,15,16], a hypothesis on the fundamental role of the cell membrane potential was introduced by Cone. Both the Ambrose et al. experimental results [19] and Cone’s hypothesis were confirmed in the following years. Recently, the fundamental role of the membrane electric potential has been highlighted in the control of the critical cell functions, such as proliferation, migration, and differentiation [20,21,22,23]. Moreover, intercellular communication has been shown to be able to modify the membrane electric potential [2]. Consequently, the fundamental role of the membrane electric potential has been pointed out in relation to the functions of cells.

Here, we have pointed out how life can be related to thermo-electric effects, occurring in the interaction between a cell and its environment, by introducing a non-equilibrium thermodynamic approach.
